# Complete Genome Sequencing of Two Equine Influenza A(H3N8) Virus Strains Isolated in Kazakhstan

**DOI:** 10.1128/genomeA.00574-18

**Published:** 2018-06-28

**Authors:** Yerbol Burashev, Vitaliy Strochkov, Kulyaisan Sultankulova, Mukhit Orynbayev, Abylay Sansyzbay, Nurlan Sandybayev, Sergazy Nurabayev, Irina Savitskaya, Daniel L. Rock, Edan R. Tulman

**Affiliations:** a Research Institute for Biological Safety Problems (RIBSP), Gvardeyskiy, Kazakhstan; b Faculty of Biology and Biotechnology, Al-Farabi Kazakh National University, Almaty, Kazakhstan; c Department of Pathobiology, College of Veterinary Medicine, University of Illinois at Urbana-Champaign, Urbana, Illinois, USA; d Department of Pathobiology and Veterinary Science and Center of Excellence for Vaccine Research, University of Connecticut, Storrs, Connecticut, USA

## Abstract

Here, we report the complete genome sequencing of strains A/equine/Kostanay/9/2012(H3N8) and A/equine/LKZ/9/2012(H3N8) of the equine influenza virus belonging to Florida sublineage, clade 2. The strains were isolated in 2012 in the northern and southern regions of Kazakhstan, respectively.

## GENOME ANNOUNCEMENT

Equine influenza virus (EIV) is a causative agent of acute respiratory disease in horses. It is classified antigenically as an influenza A virus belonging to the *Orthomyxoviridae* family that contains eight segmented negative-polarity RNA molecules (1). The first EIV (A/equine/Prague/56) was isolated in Europe in 1956 and belonged to the H7N7 subtype; however, H7N7 has not been isolated from horses for over 20 years (2). The second subtype isolated from horses, A(H3N8), was initially isolated in 1963 as an avian influenza virus and continues to circulate and cause large-scale epizootics in horses (3–6). Even Australia—previously free of EIV—experienced a large outbreak in 2007 (7). Major epizootics of EIV in Kazakhstan, Mongolia, and China took place in 2007 to 2008 and again in 2012 when the strains A/equine/Kostanay/9/2012(H3N8) and A/equine/LKZ/9/2012(H3N8) were isolated (8). Phylogenetic analysis of these isolates and another from Kazakhstan in 2012 has shown that they all belong to the Florida sublineage, clade 2 (Fc2) (9; Y Burashev, V Strochkov, K Sultankulova, M Orynbayev, unpublished data). To provide comprehensive sequence data from multiple 2012 EIV isolates from different regions in Kazakhstan, all genomic segments of strains A/equine/Kostanay/9/2012(H3N8) and A/equine/LKZ/9/2012(H3N8) were sequenced.

In the course of isolation, A/equine/Kostanay/9/2012(H3N8) and A/equine/LKZ/9/2012(H3N8) underwent five passages in 10-day-old chicken embryos. Viral RNA was extracted using the QIAamp viral RNA extraction kit (Qiagen) according to the manufacturer’s instructions. All eight genes were amplified using SuperScript One-Step reverse transcriptase PCR (RT-PCR) kits with Platinum Taq (Invitrogen SRL). For each gene amplification, three to five pairs of primers were selected with the help of the online program Primer-BLAST (http://www.ncbi.nlm.nih.gov/tools/primer-blast) to produce amplicons of 500 to 700 bp each and overlapped each other by approximately 100 bp. Sequencing was performed using a 16-capillary genetic analyzer AB3130xl automatic sequencer (Hitachi Applied Biosystems) with use of the BigDye Terminator version 3.1 cycle sequencing kit (ABI, Foster City, CA, USA). Chromatograms were processed with the use of Sequencer version 5 (Gene Codes Corp.) and BioEdit version 7.2.5 (http://www.mbio.ncsu.edu/BioEdit/bioedit.html) for sequence assembly and alignment. Genome sequencing of A/equine/Kostanay/9/2012(H3N8) and A/equine/LKZ/9/2012(H3N8) yielded sequences of all eight genomic segments, including polymerase basic 2 (PB2), polymerase basic (PB1), polymerase acidic (PA), hemagglutinin (HA), nucleoprotein (NP), neuraminidase (NA), matrix (M), and nonstructural (NS). Despite having been isolated simultaneously (although at a distance of about 2,000 km apart), the strains under study revealed differences in the following positions: PB2-C1952A, PB1-A967G, and PA-G30A, G168T, and T1250C.

Complete genome sequencing of EIV A(H3N8) strains A/equine/Kostanay/9/2012(H3N8) and A/equine/LKZ/9/2012(H3N8) belonging to Fc2 indicates the influence of spatial factors on the evolution of EIV isolated within 1 year and from outbreaks across Kazakhstan.

### Accession number(s).

The complete genome sequences of EIV A(H3N8) strains A/equine/Kostanay/9/2012(H3N8) and A/equine/LKZ/9/2012(H3N8) isolated from sick horses in the northern and southern regions of Kazakhstan are deposited in GenBank under the accession numbers listed in Table 1.

**TABLE 1:** Accession numbers for the complete genome nucleotide sequences of EIV A(H3N8) strains A/equine/Kostanay/9/2012(H3N8) and A/equine/LKZ/9/2012(H3N8)

Strain	Accession no. by genes in GenBank^a^							
	PB2	PB1	PA	HA	M	NP	NA	NS
A/equine/Kostanay/9/2012(H3N8)	MH173059	MH173058	MH173057	KP202380	KP202384	MH173056	KP202376	KP202388
A/equine/LKZ/9/2012(H3N8)	MH173322	MH173321	MH173320	KP202378	KP202382	MH173319	KP202374	KP202386

a PB2, polymerase basic 2; PB1, polymerase basic; PA, polymerase acidic; HA, hemagglutinin; M, matrix; NP, nucleoprotein; NA, neuraminidase; NS, nonstructural.
